# Cutaneous *Aspergillus fumigatus* infection in a Newborn

**Published:** 2019-04-25

**Authors:** Cara M. Barber, Matthew P. Fahrenkopf, Megan L. Dietze-Fiedler, Jenny L. Nguyen, John A. Girotto

**Affiliations:** ^a^Michigan State University College of Human Medicine, Grand Rapids; ^b^Spectrum Health/Michigan State University Plastic Surgery Residency, Grand Rapids; ^c^Pediatric Plastic Surgery and Dermatology, Helen DeVos Children's Hospital, Grand Rapids, Mich

**Keywords:** premature, fungal, cutaneous, *Aspergillus fumigatus*, neonatal

## DESCRIPTION

A preterm infant was born at 23 weeks 2 days, via emergency cesarean section, secondary to maternal hemorrhage and suspected placental abruption. On day 9 of life, she developed a spontaneous cutaneous eschar on the right flank. This was punch biopsied and later grew *Aspergillus fumigatus*.

## QUESTIONS

1. What is the incidence of neonatal fungal infections?
2. What is the differential diagnosis?
3. What are the types of *Aspergillus* infection in newborns?
4. How do you treat neonatal fungal infections?

## DISCUSSION

The neonatal period is a risky time for opportunistic pathogens to occur, especially invasive fungal infections, given the immaturity of host defenses, particularly impaired phagocytosis.^1^^,^^2^ Newborns are at the most risk for acquiring an infection during the first week of life when the most invasive therapies are performed.^1^ The advancements in the management of neonatal complications put many of them at risk for these infections, such as catheters, parenteral nutrition, glucocorticoids, and use of broad-spectrum antibiotics.^1^^,^^2^ Cutaneous fungal infections occur in a significant proportion of very low-birth-weight infants (12%-27%).^3^ Systemic fungal infections were once considered a rare complication (5% of low-birth-weight babies), whereas now they occur in 20% of babies weighing less than 1000 g. The mortality associated with these systemic fungal infections is high, at 50%.^4^

When dealing with neonatal infections, it is important to narrow down the differential diagnosis to avoid unwarranted treatment. The differential diagnosis for a rash on a newborn can be vast, with most being from bacterial such as *Staphylococcus aureus and Streptococcus pyogenes* and fungal causes.^5^ If a fungal infection disseminates, it can present with signs similar to bacterial sepsis, intracranial hemorrhage, or thrombosis, with temperature instability, refusal of feeds, respiratory distress, decreased perfusion, or seizures.^4^ The leading cause of fungal infections in the neonatal setting is the *Candida* spp, especially *Candida albicans*, followed by *Malassezia furfur*, and *Aspergillus* spp.^6^ *Aspergillus* spp can be found in numerous hospital environments, equipment, and the hospital air itself and is considered a rare cause for fungal infections in newborns.^2^^,^^4^^,^^7^ Identification of these pathogens may be accomplished through blood cultures, skin biopsies, antigen detection, or polymerase chain reaction.^8^

Cutaneous aspergillosis in newborns can be either primary or secondary.^2^ Primary cutaneous aspergillosis is characterized by acute inflammation, purulent abscess formation, tissue edema, and necrosis, similar to black eschars. This classification of aspergillosis is also known for its lack of involvement of organs except skin at the time of diagnosis.^2^ Secondary aspergillosis is characterized by the pathogen's hematogenous spread and maculopapular eruption, caused by thrombosis of small vessels.^2^ Hematogenous spread can quickly become disseminated *Aspergillus*, with most common distal site being the brain. Metastatic necrotic lesions have also been reported in the spleen, heart, and other organs.^2^

Treatment of fungal infections in newborns is time sensitive. These infections need to be treated aggressively, as disseminated fungal infections pose a threat to the newborn's health, with both short- and long-term consequences. Prompt removal or replacement of central catheters after a diagnosis of fungemia has been associated with decreased mortality rates and improved neurodevelopmental outcomes among survivors.^5^ Focal lesions should be surgically debrided to clean margins. Amphotericin B, namely, the liposomal and lipid complex forms, alone or with flucytosine, is the main antifungal treatment of systemic infections.^2^^,^^4^ Amphotericin B use is limited by the risks of nephrotoxicity, hepatotoxicity, thrombocytopenia, hypokalemia, and hypomagnesemia.^4^ A less potent alternative is fluconazole if the fungi is susceptible. It is well tolerated in the neonatal population and distributes well in body tissues and the central nervous system.^2^^,^^5^ Prophylactic treatment with fluconazole in extremely premature infants has only been beneficial when used in neonatal intensive care units with high prevalence rates of fungal infections.^8^ The remainder of care is supportive.

**Figure 1:**
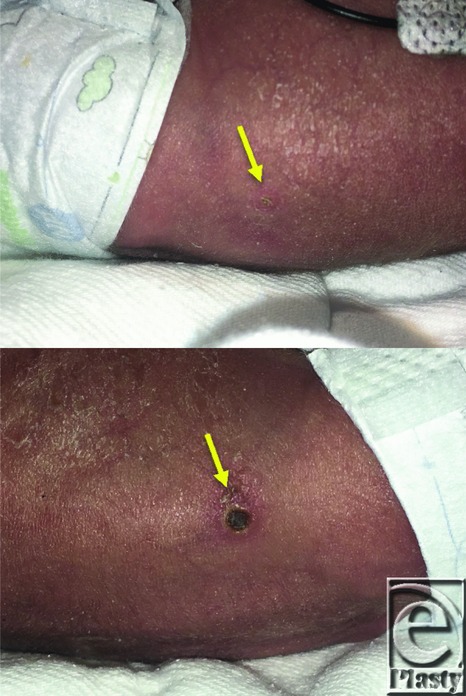
Initial appearance of cutaneous lesions on the left flank (top) and the right flank (bottom).

**Figure 2:**
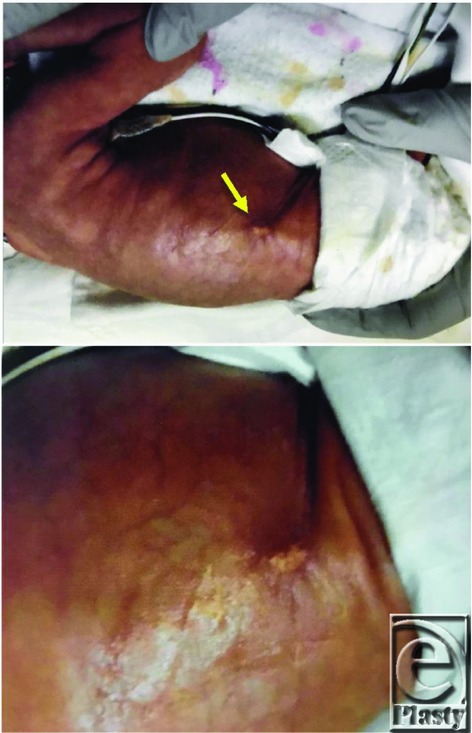
One-month postoperative appearance of the right flank lesion after wide local excision.

At birth, the patient was placed on prophylactic antibiotics (vancomycin, gentamicin, and fluconazole), had a central catheter inserted, and was started on steroids, all risk factors for developing fungal infections. Antibiotic therapy was de-escalated to antifungals (fluconazole) on day 4 of life. On day 9 of life, the right flank lesion developed (Fig 1). She was switched to vancomycin, gentamicin, and amphotericin B. Punch biopsies of the right flank lesion and a suspicious left flank lesion were done. KOH preparation demonstrated fungal elements only in the right biopsy, and PAS histochemical stains further confirmed fungal elements. Wide local excision was performed around the right biopsy site and allowed to heal secondarily (Fig 2). Systemic workup was negative for dissemination, and final tissue pathology grew *Aspergillus fumigatus*.
